# Supplementary material for: Environmental Signals Act as a Driving Force for Metabolic and Defense Responses in the Antarctic Plant Colobanthus quitensis
Source: Plants (Basel). 2022 Nov 21;11(22):3176. doi: 10.3390/plants11223176 (PMC9695728; doi:10.3390/plants11223176)
Supplement: Supplementary file 1 [file plants-11-03176-s001.zip › plants-2004977-supplementary/Supplementary/Figure S1.pdf]

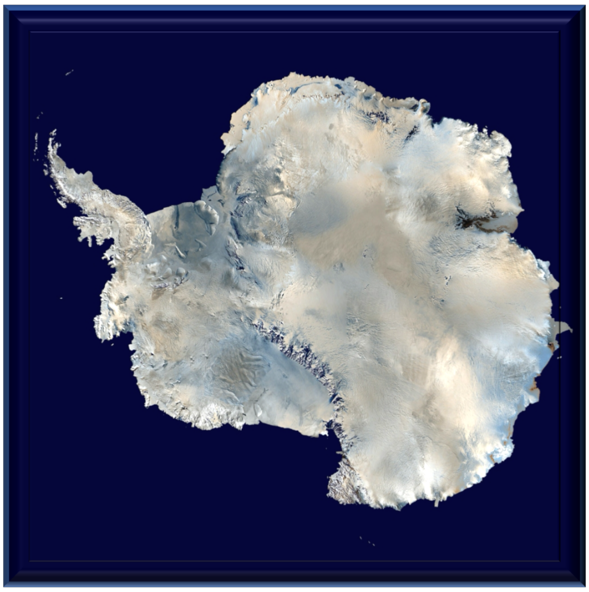

(a)

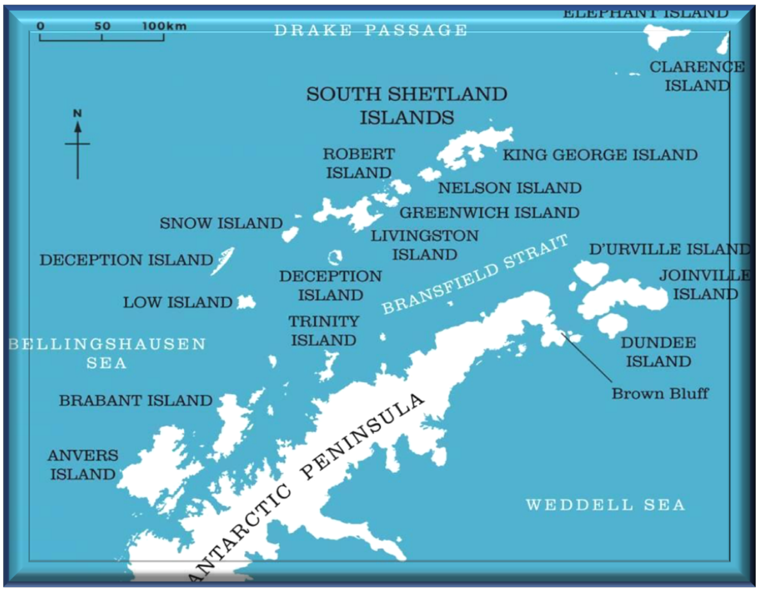
0 50 100km
DRAKE PASSAGE
ELEPHANT ISLAND
CLARENCE ISLAND
SOUTH SHETLAND ISLANDS
N
ROBERT ISLAND
KING GEORGE ISLAND
NELSON ISLAND
GREENWICH ISLAND
SNOW ISLAND
LIVINGSTON ISLAND
DECEPTION ISLAND
D'URVILLE ISLAND
DECEPTION ISLAND
JOINVILLE ISLAND
LOW ISLAND
BRANSFIELD STRAIT
TRINITY ISLAND
BELLINGSHAUSEN SEA
DUNDEE ISLAND
Brown Bluff
BRABANT ISLAND
ANVERS ISLAND
ANTARCTIC PENINSULA
WEDDELL SEA

(b)

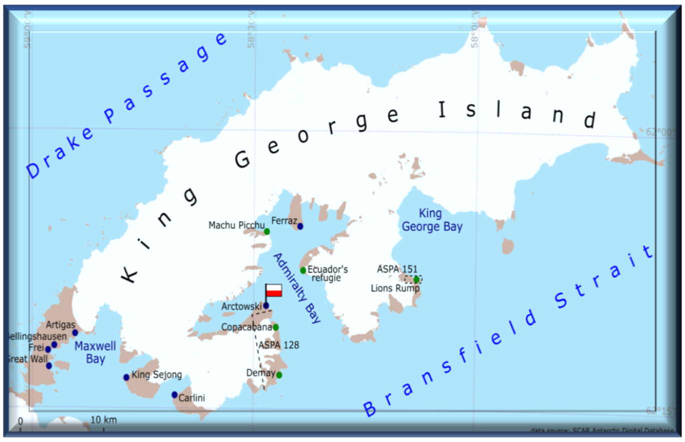
Drake Passage
King George Island
Machu Picchu
Ferraz
King George Bay
Admiralty Bay
Ecuador's refugie
ASPA 151
Lions Rump
Arctowski
Copacabana
ASPA 128
Artigas
Maxwell Bay
King Sejong
Demay
Carlini
Bransfield Strait
10 km

(c)

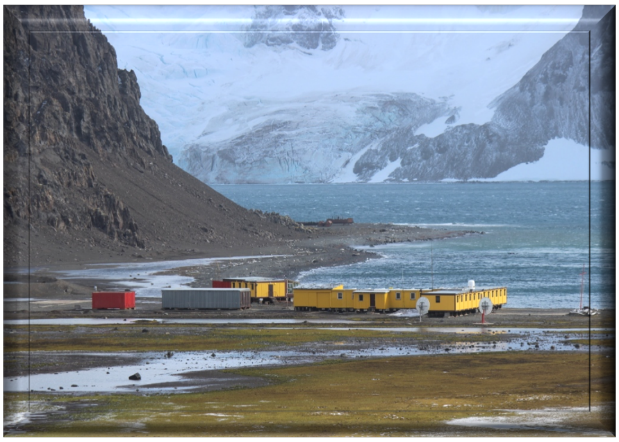

(d)

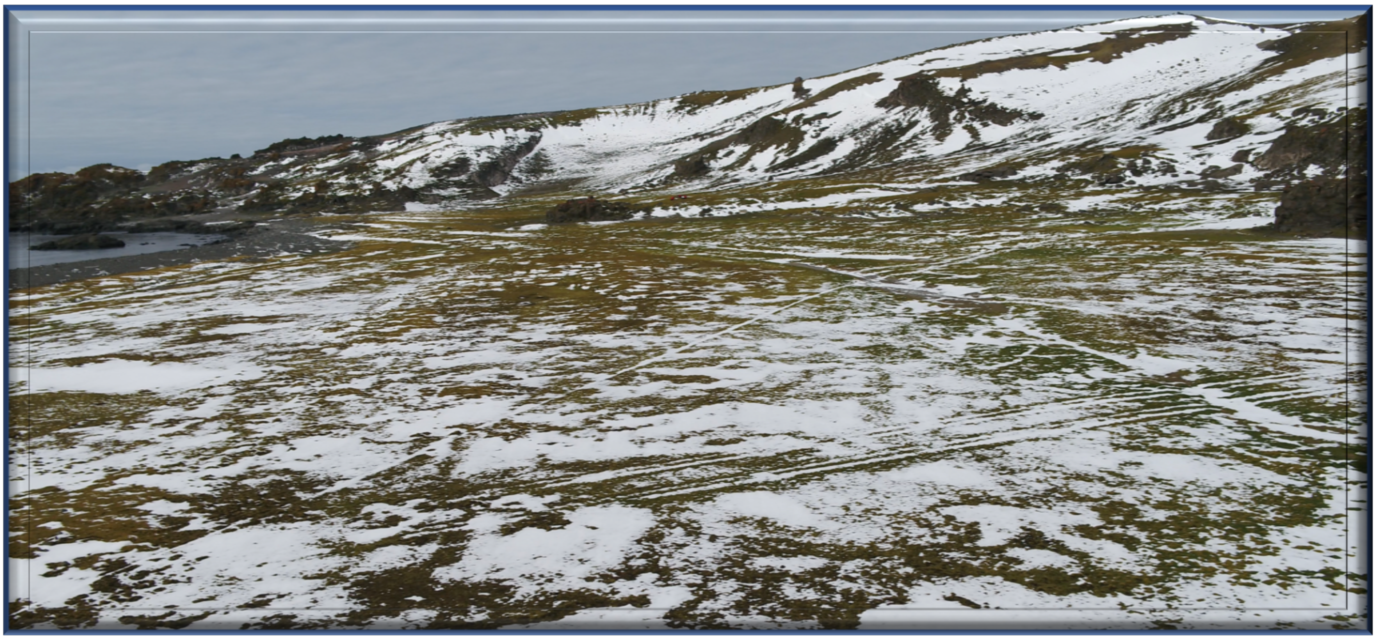

(e)

Figure S1. Sampling sites.
Panel (a) The image was taken from:
https://www.coolantarctica.com/gallery/scenic/views_of_antarctica.php
(Unlabeled free use relief map of Antarctica)
Panel (b) The image was taken from:
https://www.cruisemapper.com/ports/antarctic-peninsula-port-982
Map credit: Swoop Antarctica (https://www.swoop-antarctica.com/)
Panel (c) The image was taken from the Arctowski Polish Antarctic Station website (https://arctowski.aq/en/about-station/). The source of the figure is the SCAR Antarctic Digital Database (ADD) (https://www.scar.org/resources/antarctic-digital-database/). ADD is a free database in which the data are made available under the Creative Commons Attribution 4.0 International (CC BY 4.0) license.
Panels (d) and (e)
The images are from the personal archive of one of the authors.
