# Supplementary material for: Environmental Signals Act as a Driving Force for Metabolic and Defense Responses in the Antarctic Plant Colobanthus quitensis
Source: Plants (Basel). 2022 Nov 21;11(22):3176. doi: 10.3390/plants11223176 (PMC9695728; doi:10.3390/plants11223176)
Supplement: Supplementary file 1 [file plants-11-03176-s001.zip › plants-2004977-supplementary/Supplementary/Figure S2.pdf]

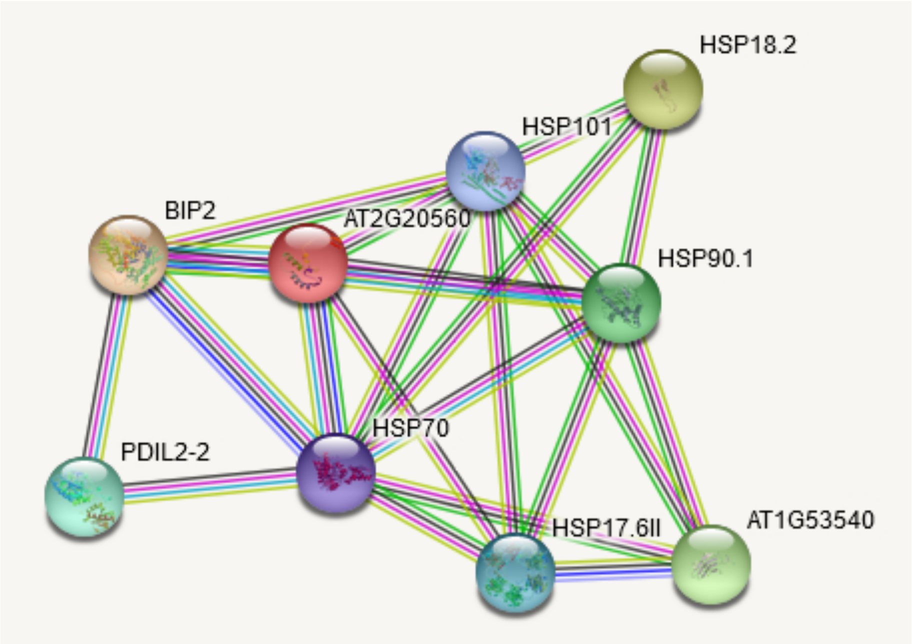

Figure S2. STRING map of interaction between down-regulated DEPs of the data set S2 vs S1, obtained at 0.7 confidence level. The colored spheres represent proteins identified with the relative TAIR code or the acronym. The interactions between the various proteins are represented by connecting lines.
